# Is the plant microbiome transmitted from pollen to seeds?

**DOI:** 10.3389/fmicb.2024.1343795

**Published:** 2024-02-13

**Authors:** Massimiliano Cardinale, Sylvia Schnell

**Affiliations:** ^1^Department of Biological and Environmental Sciences and Technologies, University of Salento, Lecce, Italy; ^2^Institute of Applied Microbiology, Justus-Liebig-University Giessen, Giessen, Germany

**Keywords:** pollen, microbiome, co-evolution, vertical inheritance, agricultural biotechnologies

## Introduction

Plants live in association with a highly diverse and abundant community of microorganisms (referred to as the “microbiome”), which actively contribute to the host's growth and fitness. Indeed, the plant is nowadays regarded as a “holobiont,” a meta-organism that includes the plant itself and its associated microbiome, seen as a single unit of evolution (Zilber-Rosenberg and Rosenberg, 2008; Vandenkoornhuyse et al., 2015). Recently, more and more studies have highlighted the possibility that microbes can be “inherited” across plant generations (Berg and Raaijmakers, 2018), thus suggesting a certain level of vertical stability (driven by co-evolution events) in the plant–microbe holobiont. This was first demonstrated in “lower plants” such as lichens and mosses (Bragina et al., 2012, 2013; Aschenbrenner et al., 2014), then in higher plants by seed transmission (Cope-Selby et al., 2017; Malinich and Bauer, 2018; Sánchez-López et al., 2018). Vertical transmission of seed microbiome is an important topic that deserves attention for especially two reasons: first, because it influences the adaptation to the habitat of the individual plants, thus directly affecting their survival chances; second, because it sheds light on the processes driving the plant–microbe holobiont co-evolution. Despite the possibility of “random colonization,” caused, for example, by a random process of microbial colonization within an individual plant or a differential colonization of the kernel fertilized by pollen grains originating from different plants (Rosenblueth et al., 2012), recent studies have demonstrated that a consistent association between plants and recurrent microbial species occurs in the seeds of several plants, irrespective of host genotype (cultivar), geographical origin, and harvesting times (Johnston-Monje and Raizada, 2011; Yang et al., 2017; Rahman et al., 2018). These are evidence of holobiont stability, which can only be the result of a natural selection process that favors beneficial microbial partners, thus generating co-evolution processes. Such co-evolutionary outcomes are already known for bacterial symbionts in various insects [aphids and *Buchnera*- Douglas (1997), aphids and *Rickettsiella*- Tsuchida et al. (2010)], as well as in other biological systems, such as marine invertebrates (O'Brien et al., 2019) and even the human gut (Van den Abbeele et al., 2011). In fact, in the evolutionary context, it is advantageous for plants to store beneficial bacteria in their seeds and transmit them to the next generation, thus enhancing both the survival chance and the fitness of their progeny. Eventually, these beneficial associations might have turned into stable holobiont traits (Rahman et al., 2018).

One aspect of this research field that still has many open questions is *how* the seed microbiome is generated, i.e., which routes are used by plants to store the beneficial microbes in their seeds. Although experimental evidence is still largely lacking, potential sources of seed-associated bacteria were suggested to be the soil (especially the rhizosphere), different plant shoot parts, meristematic tissues, and flowers (Rodríguez et al., 2018). Flowers are of special interest since the flower itself is the precursor of seeds. Flowers are composed of several tissues (e.g., pistil, anthers, pollen grains), each one hosting a specific microbiome (Junker and Alexander, 2015). In particular, the pollen was recently indicated as a potential source of seed-associated microbes (Nelson, 2018; Rodríguez et al., 2018). Indeed, the pollen is the only plant organ able to connect plant individuals that are distant from each other and not able to autonomously move to meet for reproduction. We suggest that the pollen not only allows the exchange of plant genetic material from one individual to another for plant reproduction but also allows the transfer of microbes that will constitute part of the seed microbiome.

## Pollen- and seed-associated microbiome

In the last few years, the diversity, structure, and co-occurrence network of the pollen microbiome associated with different plant species, both wind-pollinated and insect-pollinated, have been investigated in a few observational studies (Ambika Manirajan et al., 2016, 2018a,b; Obersteiner et al., 2016). A remarkably vast fungal and bacterial diversity in pollen was shown, which also included new bacterial species, such as *Spirosoma pollinicola* from *Corylus avellana* (Ambika Manirajan et al., 2018b) and *Robbsia betulipollinis* from *Betula pendula* (Shi et al., 2023), and even a new genus, *Saccharibacter*, from Japanese flowers (Jojima et al., 2004). Moreover, a high level of species-specificity was shown. Interestingly, the pollination type showed a significant effect on the microbiome's assembly and structure. In comparative studies between wind-pollinated and insect-pollinated plant species (Ambika Manirajan et al., 2016), the diversity was significantly lower in the insect-pollinated pollen, suggesting a possible role of the pollinating insects as a selecting force driving a more specific and less variable microbiome on the corresponding pollen, which appeared to be higher for bacteria than for fungi (McFrederick and Rehan, 2019). However, this effect of insects on the microbial diversity in flowers was shown to be different for different insect species in the same study on floral microbiome (Wei et al., 2021); thus, more investigation is required to confirm common trajectories. Hub taxa of the microbial network that are typically associated with wind-pollinated and insect-pollinated pollens include *Methylobacterium*/*Friedmanniella* and *Rosenbergiella*, respectively (Ambika Manirajan et al., 2018a), which are also retrieved in other floral compartments such as the nectar (Vannette, 2020).

A consistent body of literature on the effects of microbes on pollen-insect (and, in general, flower-insect) interactions exists. All studies agree that the physical contact between plant flowers and visiting insects (e.g., pollinators) alters the microbiomes of both, thus clearly indicating a microbial exchange and a bi-directional effect (Ushio et al., 2015; De Vega et al., 2021; Keller et al., 2021). Although this exchange strongly affects the microbiome of both partners, the effects on insects have been prevalently studied (Adler et al., 2021), while the plant side remained relatively less investigated; at least until recently, when the role of microbes for plant health and ecology became more and more evident (Vannette, 2020; Cullen et al., 2021). It was recognized, for example, that the floral microbiome is a potential source of species that could be used in integrated pest management and/or in pollinators' protection (Álvarez-Pérez et al., 2024); on the other hand, pollinators can act as vectors of plant beneficial microbes (Hokkanen et al., 2015).

Another aspect that began to be deeply investigated is the involvement of pollen-associated microbes in medical issues, more specifically their potential role in the sensitization to the widespread pollen-originating allergic rhinitis (Obersteiner et al., 2016; Ambika Manirajan et al., 2022) or the effect of propolis used in traditional medicine. On the contrary, the possible implications of pollen as one source of seed microbiome have so far been largely neglected, i.e., the pollen–microbe–seed system has not been investigated yet, as have the microbiomes of other plant habitats (such as the rhizosphere and phyllosphere).

While pollen microbiology remained less explored in the context of the plant microbiome, much more attention was received by the seed microbiome. In the last 5 years, the body of literature about seed microbiome has notably increased; a Google Scholar search of the words “seed microbiome” resulted in just 22 hits in the year range 2005–2015, 204 in 2016–2019, and 784 in 2020–2023; however, for the same year ranges, the search for “pollen microbiome” retrieved just 2, 39, and 56 hits. Almost all studies reported that the microbes residing in the seeds are beneficial to their plant host. In the case of barley, for example, it was shown that seeds host some stable endophytic bacteria, such as *Pantoea, Paenibacillus*, and *Pseudomonas* spp., which were able to colonize the roots upon germination with exceptional and specific (Duan et al., 2023) rhizocompetence performance and showed beneficial effects including plant growth promotion and reduction of symptoms caused by the phytopathogen *Blumeria graminis* (Rahman et al., 2018). In addition, pioneering studies on unusual plants, such as cacti, demonstrated that the seed microbiome is extremely important for seed germination and seedling survival (Puente et al., 2009).

## Concepts for future research

**Figure 1:**
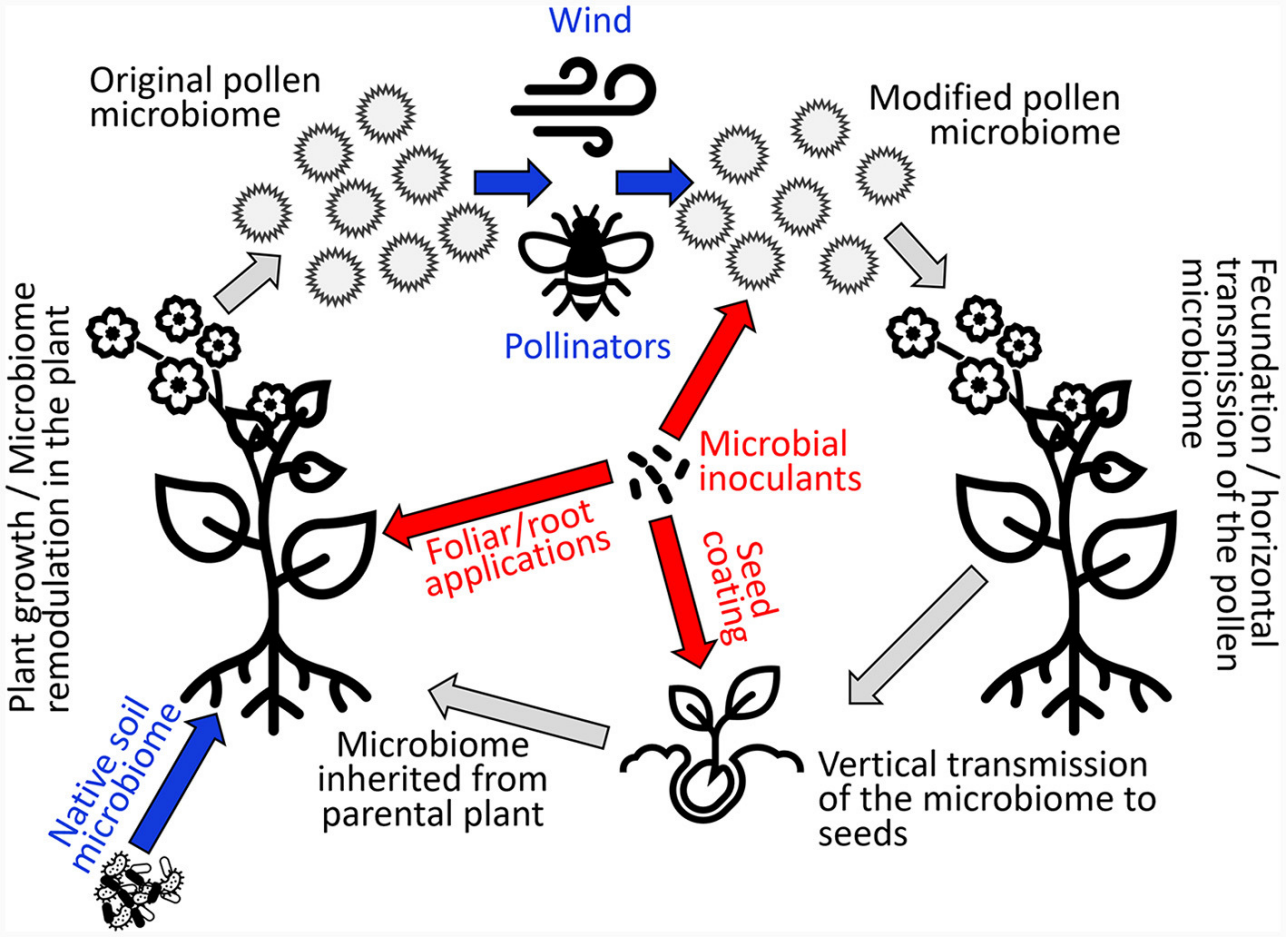
Horizontal and vertical transmission of the pollen microbiome throughout the plant cycle (black labels) and the involvement of both environmental (blue labels) and possible anthropic (red labels) factors.

We argue here that pollen microbiome research should be expanded and integrated with the better-known seed microbiome and, in general, plant microbiome data. We suggest that the pollen represents a transmission route for the seed microbiome within individuals of the same plant species. This would be a functional pathway for the plants to maintain holobiont stability across space and time through the maintenance of a healthy microbiome across plant generations. To prove this, it will be necessary to investigate whether the microbial species associated with the pollen are transmitted to the seeds of the same plant species. Considering that we now have data on the seed microbiome of many different plant species, it is possible to design specific studies to unravel the fate of pollen-vectored microbes. According to our hypotheses, the pollen microbiome and the seed microbiome will be more similar within the same plant species than between plant species, thus indicating a specific and continuous transmission of the microbiome from pollen to seeds (Figure 1). The fraction of microbial taxa shared by pollen and seeds can be regarded as the pollen-originating fraction of the seed microbiome. As a control to prove this, other compartments of the same plants could be used, where a significantly lower presence of pollen-originating taxa should be found, such as the endosphere or endorhiza. This should be especially true for insect-pollinated species, where the pollen is not transported through the air but on the body of the pollinators, which mostly visit the flowers of plants compared to other plant compartments.

To substantiate observational studies on environmental samples where the effects of soil conditions, climate, and other environmental constraints, such as habitat diversity, could mask the evidence of pollen-to-seed transmission, experiments under strictly controlled conditions will be needed. So far, only one study (to the best of our knowledge) has explored this possibility, and it confirmed the possible role of insects as vectors of microbes that shape the seed microbiome (Prado et al., 2020). Interestingly, in this study, it was also found that the seed microbial diversity was reduced by insect pollination, similar to what was observed for the pollen microbial diversity (Ambika Manirajan et al., 2016).

In future research, potential candidates for microbes to be used in specific transmission experiments can be deduced from there observations. For this, pollen inoculated with a selected bacterial strain (which could be GFP-labeled) could be used to fecundate flowers of the same species, and, after the production of seeds, these can be checked for the colonization of the inoculated bacteria. The results of these studies would notably improve our knowledge of complex plant microbiome transmission and maintenance and, finally, our understanding of holobiont ecology and evolution.

Furthermore, several aspects of pollen microbiology appear relevant in the field of agriculture. In fact, new ideas can be elaborated for the development of conceptually new techniques for bacterial inoculation, which could be used in future agricultural biotechnology applications and precision agriculture. As an example, microbial manipulation of pollen could be a complementary strategy to the seed coating because microbial inoculants delivered (directly or indirectly) on the pollen have the potential to colonize the most intimate part of the seeds in their earliest generation steps, thus being incorporated as endophytes in the mature seeds. Additionally, it is well-known that microbes associated with pollen and flowers can influence both the behavior (Russell and Ashman, 2019; Noman et al., 2020) and fitness (Dharampal et al., 2019) of insects, which means that manipulating the pollen microbiome can also improve pollination performances. As an example, it was shown that inoculating the yeast *Metschnikowia reukaufii* in flower nectars of plants, such as *Clematis akebioides* (Yang et al., 2019) and *Delphinium barbeyi* (Schaeffer et al., 2014) modified pollinators' preferences, mostly through nectar chemistry alteration and VOC production.

We hope that this opinion paper could serve as an inspirational reading to stimulate the research of pollen microbiology, which can finally integrate the knowledge of the pollen–insect/environment–seed–plant continuum (Figure 1).

## Author contributions

MC: Conceptualization, Writing – original draft. SS: Conceptualization, Writing – review & editing.
